# Research progress of nanocarriers for gene therapy targeting abnormal glucose and lipid metabolism in tumors

**DOI:** 10.1080/10717544.2021.1995081

**Published:** 2021-11-03

**Authors:** Xianhu Zeng, Zhipeng Li, Chunrong Zhu, Lisa Xu, Yong Sun, Shangcong Han

**Affiliations:** a Department of Pharmaceutics, School of Pharmacy, Qingdao University, Qingdao, China; b School of Public Health, Qingdao University, Qingdao, China

**Keywords:** Targeted nano-drug carrier, abnormal glucose and lipid metabolism, gene carrier drugs, tumors

## Abstract

In recent years, the incidence of various types of tumors has gradually increased, and it has also been found that there is a certain correlation between abnormal glucose and lipid metabolism and tumors. Glycolipid metabolism can promote tumor progression through multiple pathways, and the expression of related genes also directly or indirectly affects tumor metabolism, metastasis, invasion, and apoptosis. There has been much research on targeted drug delivery systems designed for abnormal glucose and lipid metabolism due to their accuracy and efficiency when used for tumor therapy. In addition, gene mutations have become an important factor in tumorigenesis. For this reason, gene therapy consisting of drugs designed for certain specifically expressed genes have been transfected into target cells to express or silence the corresponding proteins. Targeted gene drug vectors that achieve their corresponding therapeutic purposes are also rapidly developing. The genes related to glucose and lipid metabolism are considered as the target, and a corresponding gene drug carrier is constructed to influence and interfere with the expression of related genes, so as to block the tumorigenesis process and inhibit tumor growth. Designing drugs that target genes related to glucose and lipid metabolism within tumors is considered to be a promising strategy for the treatment of tumor diseases. This article summarizes the chemical drugs/gene drug delivery systems and the corresponding methods used in recent years for the treatment of abnormal glucose and lipid metabolism of tumors, and provides a theoretical basis for the development of glucolipid metabolism related therapeutic methods.

## Graphical Abstract

**Figure:**
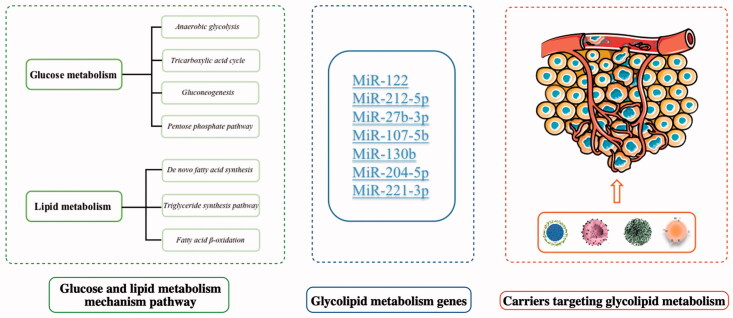

## 1. Introduction

At present, studies (Shlomai et al. 2016; Wojciechowska et al. 2016; Miao et al. 2017) have found that patients with diabetes have a higher incidence of related tumor diseases than normal people. Research data show that the risk of colorectal cancer in diabetic patients is 27% higher than that of normal people (Gonzalez et al. 2017). Thus, researchers have concluded that the occurrence of tumors is related to abnormal glucose and lipid metabolism. In the process of glucose and lipid metabolism, hexokinase and phosphofructokinase play an important role in the glycolysis process (Tao et al. 2017), and Katagiri et al. (Katagiri et al. 2017) found that the high expression level of type II hexokinase is related to the size of tumors. The degree of invasion and the nature of metastasis are significantly related to the increase in tumor recurrence rate and the overall mortality of patients.

Enzymes in various pathways of glucose and lipid metabolism (He et al. 2015) promote the process of glycolysis, and by inhibiting or promoting enzymatic activity, affect tumor growth. This is the main entry point for the current treatment of abnormal glucose and lipid metabolism. Based on this theory, constructing the corresponding chemical drug carrier, loading the corresponding inhibitor, and targeting to the corresponding site have greatly increased the therapeutic effect and efficiency of the drug.

With the deepening of genetic engineering research, gene therapy has become another widely used method of clinical treatment (Sankar & Cho 2015). In recent years, researchers have discovered that gene mutations are important factors that can induce hereditary diseases and tumors. The development of gene editing and vector technology will provide new treatment methods that can replace traditional treatment options.

Based on a series of effects of gene therapy technology and glucose and lipid metabolism on tumors, relevant gene fragments can be determined, appropriate gene carriers can be selected, and silencing RNA or interfering RNA technology can be used (Zhang & Hua 2004), and the expression of genes related to glucose and lipid metabolism can then be regulated to subsequently affect the tumor growth process. In this review, we summarize the recent research that has determined the mechanism of the related gene therapy technology for the treatment of tumors from the perspective of glucose and lipid metabolism, in order to further reveal the potential connection between abnormal glucose and lipid metabolism and tumor proliferation, metabolism, metastasis, and apoptosis. Additionally, the identification of new glycolipids, metabolism-related gene fragments, and development of new tumor-related gene therapy technologies provide summaries and assistance, and also indicate viable new prospects for glucose and lipid metabolism-related gene therapy for tumors.

## 2. Abnormal glucose metabolism and lipid metabolism

Cancer cells preferentially undergo glycolysis when sufficient oxygen is present, which is called the Warburg effect (Wu et al. 2020). Abnormal metabolism is a significant marker of cancer and can be found in many types of cancers (Pavlova & Thompson 2016). Cancer cells can produce a great deal of energy by increasing their metabolism (Stine et al. 2015), and this energy is used for their growth, the irregular growth of tumor blood vessels, and for influencing the tumor microenvironment (TME).

### 2.1. Cancer and glucose metabolism

**Figure 1.**
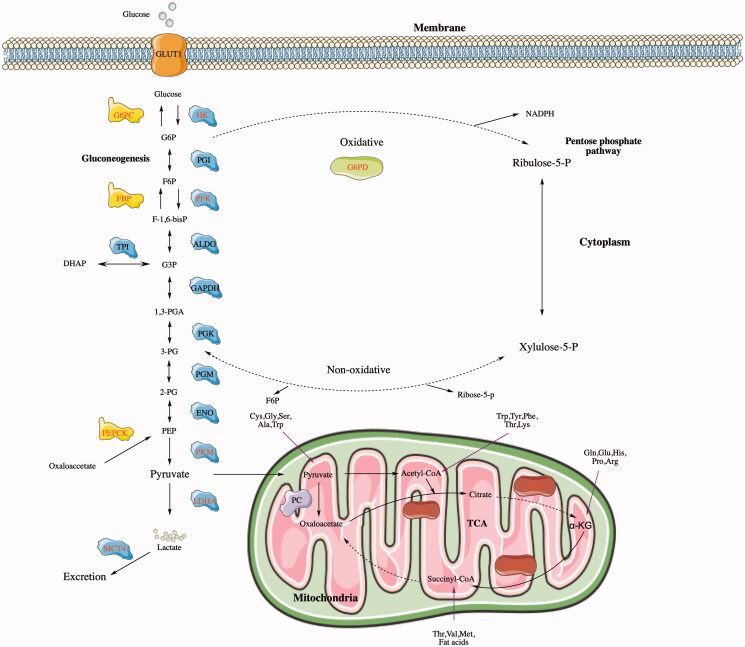
The glucose metabolism process. Glucose enters the cell via a glucose transporter (GLUT). The glycolysis process uses multiple enzymes, including HK, PFK, and PK. Gluconeogenesis affects TCA, PPP, and other processes. Three key enzymes regulate this effect in gluconeogenesis, namely, glucose-6-phosphatase-alpha (G6PC), FBP, and PEPCK. Pyruvate enters the mitochondria and generates carbon dioxide and water through the TCA pathway, which is catalyzed by three key enzymes: IDH, CS, and ketoglutarate dehydrogenase complex (KGDHC). G6PD is a key enzyme in the oxidation process of the PPP pathway, and regulates the reaction of the PPP process in the cytoplasm.

Carbohydrate metabolism is the main method of cellular capacitation (Hereng et al. 2014). In cancer cells, carbohydrate metabolism is upregulated, followed by an increase in glycolysis. Additionally, there are multiple carbohydrate metabolism pathways in cells (as shown in Figure 1), including anaerobic glycolysis, the tricarboxylic acid (TCA) cycle, gluconeogenesis, and the pentose phosphate pathway (PPP) (Zhang et al. 2020).

#### 2.1.1. Anaerobic glycolysis

Glucose is finally converted into lactate after anaerobic glycolysis, and several key glycolytic enzymes and glucose transporters (GLUTs) are involved in this process. The overexpression of key GLUTs mediates the enhancement of glucose metabolism in tumor cells (Jóźwiak et al. 2012). Thus far, 14 GLUT subtypes encoded by different genes have been identified. Different subtypes of transport enzymes possess different affinities for glucose and other hexoses, and selectively transport different sugar molecules. Among them, GLUT 1–4 are the most well-known four subtypes. GLUT1, GLUT2 (SLC2A2), GLUT3 (SLC2A3), and GLUT4 (SLC2A4) have completely different regulatory mechanisms and dynamic characteristics, yet they are all effective in maintaining glucose homeostasis in cells and organisms. Each of them performs a specific function (Thorens & Mueckler 2010). When cells uptake a large amount of glucose, it is converted into pyruvate by a reaction inside the cells. Three key enzymes, hexokinase 2 (HK2), phosphofructokinase (PFK), and isocitrate dehydrogenase (IDH), act as rate-limiting agents (Crousilles et al. 2018; Fernandes et al. 2020), and their high expression promotes the malignant development of cancer cells (O'Neal et al. 2016; Ma et al. 2019; Yang et al. 2019).

#### 2.1.2. TCA cycle

The TCA is the main pathway of cellular oxidative phosphorylation, which meets the requirements of bioenergy, biosynthesis, and redox balance (Payen et al. 2020). Although it was previously believed that cancer cells would bypass the TCA cycle and use aerobic glycolysis, emerging evidence suggests that certain cancer cells, especially those with abnormal expression of oncogenes and tumor suppressor factors, rely heavily on the TCA cycle to generate energy and synthesize macro molecules (Anderson et al. 2018). Cancer cells maintain their high proliferation rate and energy requirements through metabolic recombination. The TCA cycle is a central metabolic hub necessary for the production of ATP and the provision of precursors used in many biosynthetic pathways (Nazaret et al. 2009). Therefore, dysregulation of the TCA circulation flux is often observed in cancer. The mutations of several enzymes in the TCA cycle in human tumors, e.g. aconitase, isocitrate dehydrogenase 1 (IDH1), fumarate hydratase, and succinate dehydrogenase have shown that there is a direct link between this metabolic pathway and the occurrence of cancer (Jimenez-Morales et al. 2018). In addition, it has also been shown that changing the expression or activity of these enzymes can promote the metabolic adaptation of cancer cells (Ciccarone et al. 2017).

#### 2.1.3. Gluconeogenesis

Gluconeogenesis can generate free glucose from non-carbohydrate carbon substrates (such as glycerol, lactic acid, pyruvate, and glycogenic amino acids). Although it is less studied than catabolic glycolysis or oxidative phosphorylation (OXPHOS), this anabolic pathway plays the same role in controlling the aerobic glycolysis of cancer cells (Seenappa et al. 2016). The complete pathway consists of 11 enzyme-catalyzed reactions, of which there are 7 reactions that are the opposite steps of glycolysis, and 3 reactions that are not involved in gluconeogenesis: (i) the conversion of pyruvate to phosphoenolpyruvate, which is determined by the reaction that catalyzes pyruvate carboxylase (PC) and phosphoenolpyruvate carboxykinase (PEPCK); (ii) the catalyzation of the conversion of fructose-1,6-diphosphate to fructose-6-phosphate by fructose-1,6-bisphosphatase (FBPase); (iii) the catalyzation of the conversion of glucose-6-phosphate to glucose by glucose-6-phosphatase (G6Pase) (Icard et al. 2019). PEPCK, FBPase, and G6Pase are the key enzymes that control the gluconeogenesis flux, thereby affecting glycolysis, the TCA cycle, the PPP and other branched metabolic pathways (serine biosynthesis, glycogen health, gluconeogenesis, and glutamine decomposition) (Kang et al. 2016; Icard et al. 2019).

Cancer cells display a high rate of glycolysis in the presence of oxygen to promote proliferation. Gluconeogenesis is the reverse pathway of glycolysis, and it can antagonize the aerobic glycolysis in cancer via three key enzymes: PEPCK, FBPase, and G6Pase (Vincent et al. 2015; Wang & Dong 2019). Recent studies have revealed that in addition to metabolic regulation, these enzymes also play a vital role in signaling, proliferation, and the designation of cancer stem cell (CSC) tumor phenotypes. Multifaceted regulation of PEPCK, FBPase, and G6Pase through transcription, epigenetics, post-translational modification, and enzymatic activity can be observed in different cancers (Leithner et al. 2015; Montal et al. 2015).

#### 2.1.4. Pentose phosphate pathway (PPP)

The pentose phosphate pathway (PPP), also known as the hexose monophosphate bypass or phosphogluconate pathway, branches off from glycolysis when the first step is completed (Stincone et al. 2015). Under the catalysis of hexokinase, glucose-6-phosphate (G6P) is consumed as a main substrate. The PPP assists glycolytic cancer cells so that their anabolic needs are met and they are resistant to oxidative stress. Recently, it has been shown that some neoplastic lesions developed and then promoted the flux of glucose to the PPP (Patra & Hay 2014). Glucose-6-phosphate dehydrogenase (G6PD) regulates the rate of the PPP by catalyzing an irreversible step. The expression level of G6PD is different in various breast cancer subtypes, and is positively correlated with poor prognosis of patients (Pu et al. 2015).

### 2.2. Cancer and lipid metabolism

**Figure 2.**
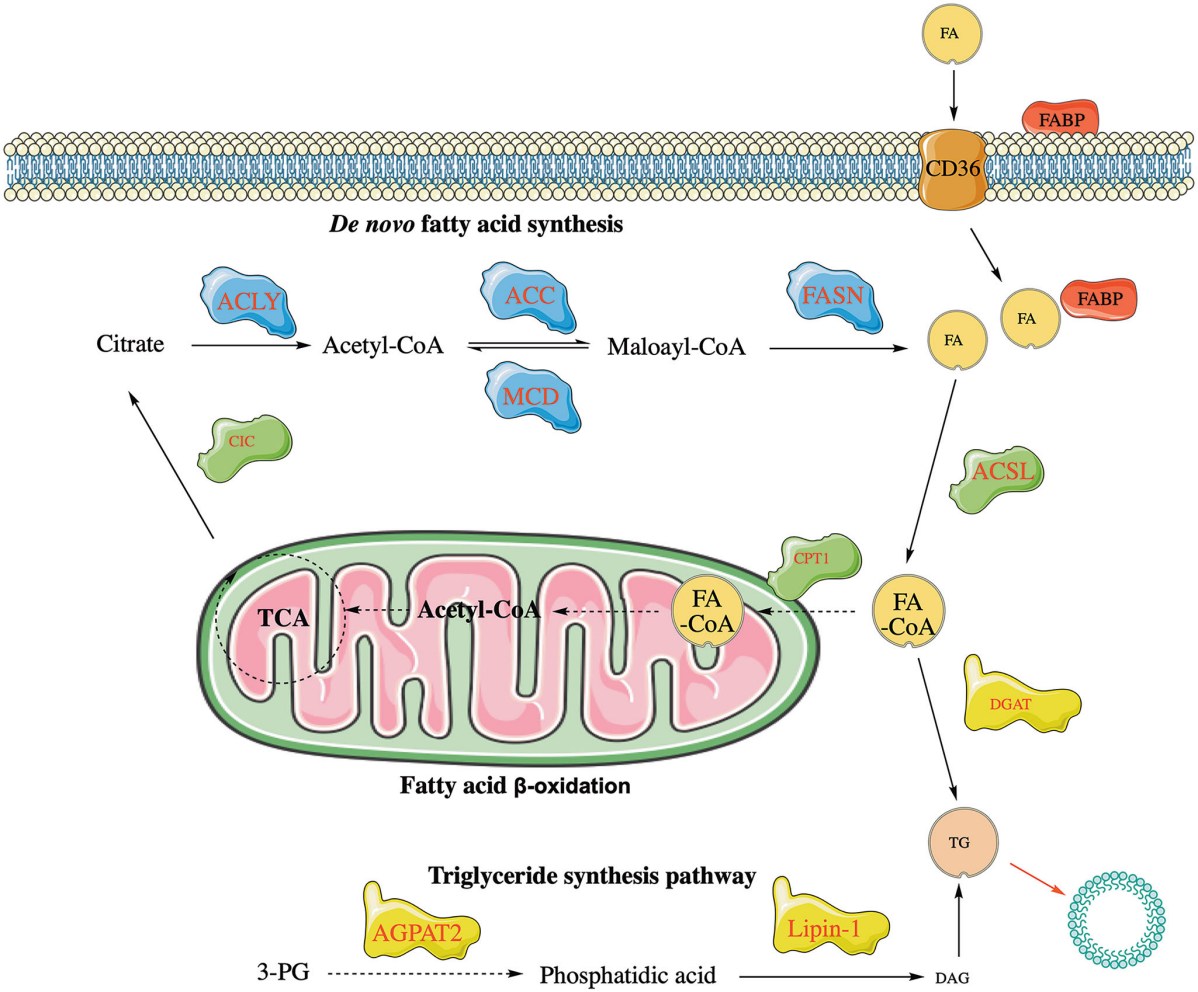
The lipid metabolism process. FA enters the cell through CD36. FA is catalyzed by the ACSL enzyme in the cell to generate FA-CoA, which is a precursor of acetyl-CoA and is used as a substrate in the TCA reaction in the mitochondria. The role of FA-CoA in DGAT is to generate TG and secrete lipid droplets to store energy.

Decades ago, researchers discovered that tumor cells can synthesize lipids in the same manner as normal cells (Medes et al. 1953). Since then, studies have also found that abnormal increases in lipid metabolism have become an important hallmark of cancer (Santos & Schulze 2012). Lipid metabolism assays showed that compared with normal cells, cancer cells increased the expression of ATP-citrate lyase, acetyl coenzyme A (acetyl-CoA) carboxylase, and fatty acid (FA) synthase that is involved in de novo lipid synthesis (Bort et al. 2020). Lipid metabolism mainly includes de novo fatty acid synthesis, the triglyceride synthesis pathway, and fatty acid β-oxidation, which subsequently affect the proliferation, metabolism, and metastasis of cancer cells (as shown in Figure 2) (Vander Heiden et al. 2009).

#### 2.2.1. De novo fatty acid synthesis

The synthesis of FAs is an essential cellular process that uses glutamine or glucose as building blocks (Rohrig & Schulze 2016; Min & Lee 2018). Citrate is a major intermediate product produced by the TCA cycle that produces FAs through the action of several key enzymes, such as ATP citrate lyase (ACLY) and fatty acid synthase (FASN) (Lee et al. 2015). ACLY converts citrate into acetyl-CoA, which is an important enzyme that can link carbohydrates and lipid metabolism by generating acetyl-CoA from citric acid, thereby achieving the biosynthesis of fatty acids (Feng et al. 2020). Acetyl-CoA carboxylase (ACC) catalyzes the formation of malonyl-CoA, which is an important substrate and key regulatory molecule for fatty acid synthesis in adipose tissue (Choosangtong et al. 2015). Adenosine monophosphate (AMP)-activated protein kinase phosphorylates and inhibits ACC, which indirectly inhibits the synthesis of fatty acid (Lepropre et al. 2018). FASN uses malonyl-CoA as a substrate to synthesize the final form of fatty acids. It has been found that FASN is associated with progression in a variety of cancers, and hence, it is an important target for cancer therapy (Jones & Infante 2015; Menendez & Lupu 2017).

#### 2.2.2. Triglyceride synthesis pathway

Triglyceride is one of the main forms of fatty acid storage and transport in the body (Alves-Bezerra & Cohen 2017), and it is synthesized via two main pathways. One is to convert 3-phosphotriglyceride, which is a substrate of phosphatidic acid, and subsequently generate diacylglycerol, the precursor to triglyceride (TG) under the catalysis of lipin-1, and finally to produce TG. The other pathway is the stepwise reaction of fatty acids to generate TG (Coleman et al. 2000; Zhao et al. 2020). The resulting lipid droplets are used for energy expenditure and/or storage (Haemmerle et al. 2002).

#### 2.2.3. Fatty acid β-oxidation

Fatty acid β-oxidation occurs in mitochondria, and in the first step, FAs enter the mitochondria in the form of FA-CoA. FA-CoA reacts to generate acetyl-CoA, which is used as a substrate in the TCA process (Orlando et al. 2019). Like other metabolic pathways related to cancer, fatty acid oxidation (FAO) is also changed in various human malignancies (Carracedo et al. 2013; Currie et al. 2013). Cancer cells rely on the FAO process for proliferation, survival, drug resistance, and metastasis. In the FAO process, cancer-related immune cells and other host cells are reprogrammed, which increases immunosuppression and affects the TME (Qu et al. 2016; Ma et al. 2018).

## 3. Genes that regulate glucose and lipid metabolism

Reprogramming of the metabolism is a hallmark of cancer, and we summarized the glucose or lipid metabolism pathways. Next, we will summarize and describe the regulation of genes from two aspects. One is to discuss the regulation pathways of genes related to glucose and lipid metabolism. Another is to describe these glucolipid metabolism-related genes in the process of tumor growth, providing some potential targets and exploration directions for cancer therapy.

### 3.1. MiR-122

MiR-122 is an miRNA that has been studied in detail, and was previously found to be an miRNA specifically expressed in the liver (Thakral & Ghoshal 2015). However, it has also been discovered that miR-122 is not only highly expressed in the liver, but it is also involved in other processes, such as spermatogenesis. It has been shown that miR-122 is useful because it can be used for the prediction and detection of liver cancer, breast cancer, and other cancers (Esau et al. 2006). The regulatory mechanism and research potential of miR-122 in glucose and lipid metabolism will be described as follows (Bandiera et al. 2015). MiR-122 is highly secreted by cancer cells, highly expressed in liver cancer, and is involved in multiple metabolic pathways.

Li et al. found that the occurrence of liver cancer is related to the mutation of some tumor suppressor genes, and some of them are associated with the overexpression of apolipoprotein B mRNA editing enzyme subunit 2 (APOBEC2). However, the expression of miR-122 is negatively correlated with the expression of APOBEC2. These results suggest that miR-122 can specifically bind to the 3′ untranslated region (3′ UTR) of APOBEC2 mRNA to inhibit its expression (Li et al. 2019). Fong et al. showed that cancer cells specifically secrete high levels of miR-122 into extracellular vesicles (EVs), such as breast cancer (Fong et al. 2015). MiR-122 suppresses glucose metabolism by downregulating the pyruvate kinase (PKM). Among genes that control glucose metabolism, miR-122 significantly affected PKM2, citrate synthase (CS), and GLUT1, which was consistent with the downregulation of PKM2. This suggests that miR-122 affected glucose metabolism through the PKM, CS, and GLUT1 pathway. The overexpression of miR-122 inhibits the glucose metabolism pathway, and although it increases the metastatic ability of cancer cells, it ultimately suppresses tumor growth (Hsu et al. 2012).

### 3.2. MiR-212-5p

Studies have found that miR-212-5p is involved in the activities of various organs and is also necessary to support the biological activities of some cells (Lin et al. 2018; Deng et al. 2019). MiR-212-5p inhibited lipid synthesis and accumulation by targeting fatty acid synthetase and stearoyl-CoA desaturase-1. In Lu et al.’s study, co-transfection results showed that miR-212 regulated fatty acid synthetase and sterol regulatory element binding factor 1 by targeting SIRT2, which was found to increase the lipid content in mammary epithelial cell lines (Lu et al. 2020). Guo et al. found that leucine deficiency led to lipid loss via inhibition of the expression of fatty acid synthetase such that miR-212-5p specifically binds to FAS 3’UTR. Further studies showed that miR-212-5p also can bind to the 3’UTR of stearoyl-CoA desaturase-1. Overexpression of miR-212-5p impedes the synthesis of lipid intermediates and ultimately reduces lipid accumulation (Liang et al. 2013; Guo et al. 2017).

MiR-212-5p plays an important role in the regulation of metastasis and invasion of various cancer cells. The expression of miR-212-5p was significantly increased in colorectal cancer. Further studies showed that the expression of miR-212-5p was negatively correlated with Drosophila mothers against decapentaplegic 4 (SMAD4), and prevented metastasis and invasion of colorectal cancer cells. With the downregulation of miR-212-5p, the metastasis of colorectal cancer cells significantly decreased (Huang et al. 2016). In addition, the targeting of sirtuin 2 (SIRT2) by miR-212-5p is important because it inhibits the metastasis and proliferation of colorectal cancer cells (Du et al. 2020). The same inhibitory effect on proliferation was also found in HepG2 hepatocellular carcinoma cells by downregulating suppressor of cytokine signaling 5 (SOCS5) (Han et al. 2020).

### 3.3. MiR-27b-3p

MiR-27b-3p is a key regulatory factor of lipid metabolism that can directly control critical lipid genes and subsequently affect lipid metabolism. For many years, targeting peroxisome proliferator-activated receptor γ (PPAR gamma) has been considered to be one of the main mechanisms by which miR-27b-3p regulates lipid metabolism. MiR-17b-3p can also suppress lipid metabolism-associated factors other than PPAR gamma (Wang et al. 2020). MiR-27b-3p promotes tumor proliferation and mitigates drug resistance in tumor cells. The role of miR-27b-3p in the growth, decay, and metastasis of tumor cells was detected by corresponding analytical methods. In gastric cancer (GC), the silencing of miR-27b-3p significantly inhibits the metastasis and invasion of GC cells, and reduces GC cell viability. The downregulation of miR-27b-3p was a method used to accurately target GSPT1, and it subsequently alleviated the malignant behavior of gastric cancer by abnormal DNA methylation ( Zhang et al. 2019; Jiang et al. 2020; Li & Guo 2020; Shen et al. 2020; Wang et al. 2020). Chemotherapy resistance is one of the major obstacles in the treatment of cancer. Downregulation of miR-27b-3p indirectly reverses the tumor growth process, but it also affects the chemical sensitivity of oral squamous cell carcinoma (OSCC) and enhances the sensitivity of OSCC cells to cisplatin (Han et al. 2020; Ma et al. 2020). In gastric cancer, miR-27b-3p inhibited the viability of human gastric carcinoma cell lines by targeting vascular endothelial growth factor C (VEGF-C). Cui et al. found that in the established heterotopic transplantation model, the combination of miR-27b-3p and lipoteichoic acid significantly inhibited tumor growth compared with either alone (Cui et al. 2021).

### 3.4. MiR-107-5b

MiR-107-5b is an important regulatory gene related to the distribution of blood glucose and lipids. In the early stage of obesity, the distribution of lipids and sugars as well as some hormones that regulate glucose and lipid metabolism have undergone significant changes. The expression of some regulatory genes has also undergone significant changes, including miR-107-5b (Deiuliis 2016). The downregulated expression of miR-107-5b decreases glucose and lipid metabolism. Under the influence of a high-glycolipid diet, the expression of related genes in mouse adipocytes was significantly changed, such as the upregulation of miR-107-5b and the downregulation of miR-125a-5p, which indirectly revealed that miR-107-5b and other genes were affected by the changes in nutrients and other external factors.

As a primary regulator of lipid and glucose metabolism (Youssef et al. 2020), miR-107-5b is also involved in the formation of cholesterol. Studies have found that miR-107-5b can affect the pathogenesis of cholesterol gallstones (Qian et al. 2021). MiR-107-5p regulates tumor proliferation and invasion, and abnormal expression of miR-107-5b has been found in a variety of human tumors and is involved in multiple stages of tumor progression. A comparison of normal endometrial tissues with endometrial carcinoma indicated that the expression of miR-107-5b in endometrial carcinoma is significantly higher than that in normal tissues. Blocking miR-107-5p can directly affect the proliferation and metastasis of endometrial carcinoma cells (Bao et al. 2019). The study also found that the expression of miR-107-5p was significantly decreased in non-small cell lung cancer tissues and non-small cell lung cancer cell lines. MiR-107-5p inhibits tumor invasion and proliferation by targeting epidermal growth factor receptor (EGFR), and ultimately suppressing tumor growth (Wang et al. 2017). These studies reveal that miR-107-5p can be used as a potential diagnostic factor and a target for the inhibition of tumor proliferation and metastasis. In addition to the upregulation of tumor expression, miR-107-5p was also highly expressed in acute aortic dissection (AD) (Wang et al. 2020), and exhibited an inhibitory effect in acute AD.

### 3.5. MiR-130b

Although abnormal expression of miR-130b has been detected in a variety of cancers (Yu et al. 2015; Mu et al. 2020), the mechanism of action of miR-130b has not been clearly elaborated until now. MiR-130b regulates the metabolism of nutrients and also participates in multiple growth processes of tumors. MiR-130b can regulate metabolism-related pathways, including fatty acid degradation, glycolipid metabolism, and other pathways (Assmann et al. 2020). It was found that Xiangsha Liujunzi decoction regulates cholesterol metabolism through long-chain non-coding miR-130b, which ultimately affects lipid accumulation. miR-130b regulates the cholesterol metabolism process mediated by PPAR gamma to decrease lipid deposition in the liver (Jiang et al. 2020; Liu et al. 2020). In addition, the miR-130 family is an important gene that regulates the progression of cancer. Several studies have shown that miR-130b is associated with the growth, blood vessel growth, metastasis, and proliferation of a variety of tumor cells. For example, miR-130b was found to act in a potential tumor network that negatively regulates hematopoietically-expressed homeobox protein (HHEX) expression. After downregulation of HHEX expression, metastasis, invasion, and proliferation of breast cancer cells was significantly higher than those of normal cell lines (Zhang et al. 2020). The same regulatory effect was also observed in cervical cancer, where the increase in miR-130b-5p (miR-130b-5p is a passenger strand of miR-130b) in cervical cancer stem cells downregulated ETS-like gene 1 (ELK1) expression. Enhancing miR-130b-5p or silencing ELK1 inhibited the self-renewal ability and tumor volume growth of cervical cancer stem cells, and promoted cell apoptosis (Huang & Luo 2021). However, opposite views exist. In lung adenocarcinoma tissues, the upregulation of miR-130b also promoted cell metastasis and invasion (Kim et al. 2021).

In addition to its regulatory role in cancer cells, miR-130b is also involved in the regulation of normal human cells. For example, miR-130b can inhibit the proliferation of myoblasts and the differentiation of corresponding stem cells. miR-130b plays a key role in muscle replacement (Wang et al. 2021), and miR-130b modulates tumor progression and increases tumor sensitivity radiotherapy and chemotherapy. Inoue’s study demonstrated that increased miR-130b expression in clinical oropharyngeal squamous cell carcinoma resulted in significantly longer progression-free survival and overall survival (Inoue et al. 2021). Clinically, chemical drugs such as cisplatin face huge drug resistance barriers in the treatment of gastric cancer and other solid tumors, and the therapeutic effect is greatly reduced. We know that the high expression of cytidine monophosphate kinase 1 (CMPK1) is also closely related to the therapeutic effect of 5-fluorouracil (5-FU). MiR-130b, a key epigenetic regulator of CMPK1, can downregulate CMPK1 and increase patient sensitivity to 5-FU in the treatment of gastric cancer (Hashimoto et al. 2020; Wang et al. 2020; 2020; Chu et al. 2021). There is sufficient evidence that miR-130b can be used as a potential target for tumor growth inhibition and new therapeutic approaches (Wang et al. 2020).

### 3.6. MiR-204-5p

MiR-204-5p plays a significant regulatory role in cancer, especially in the metabolism of fat, which includes adipocyte differentiation and adipokine metabolism, and this mechanism has a certain associated effect on the growth and proliferation of tumor cells. MiR-204-5p is a promoter of lipid synthesis, and studies have shown that miR-204-5p was highly expressed in mammary epithelial cells, and regulated lipid synthesis without affecting the proliferation of mammary epithelial cells. The overexpression of miR-204-5p significantly increased the number of signaling molecules in the lipid synthesis pathway. MiR-204-5p regulates lipid synthesis by targeting SIRT1, and the two are negatively correlated (Zhang et al. 2020). It was also found that miR-204-5p inhibited lipogenesis by inhibiting adipose stem cell differentiation, and bioinformatics analysis revealed that miR-204-5p is a potential target for the regulation of lipogenesis (Li et al. 2020). In addition to the regulation of lipid metabolism, miR-204-5p plays a role in the process of glycolysis by targeting myosin heavy chain 9 (MYH9). After the knockout of MYH9, the glycolysis of tumor cells in the absence of oxygen was inhibited, which was ultimately manifested as the inhibition of tumor growth by regulation of miR-204-5p (Fang et al. 2020).

MiR-204-5p plays an important biological role in a variety of tumors and affects the progression of tumors. Studies have found that there is a negative correlation between the expression of high mobility group protein A2 (HMGA2) and miR-204-5p, and the expression of HMGA2 affects tumor volume and tumor progression stage. Comparison of gene expression results between tumor tissues and para-cancerous tissues showed that the expression of miR-204-5p was significantly downregulated within tumor tissues. According to this result, the proliferation and metastasis of tumor cells were inhibited by the knockout of HMGA2 or the upregulation of miR-204-5p expression (Zhang et al. 2021). In gastric cancer, small nucleolar RNA host gene 4 (SNHG4) knockout upregulated the expression of miR-204-5p, resulting in an inhibitory effect on the growth of gastric cancer cells. Compared with normal gastric tissues, the expression of miR-204-5p was significantly decreased in gastric cancer tissues, and thus, SNHG4 could be used as a potential target for future treatment of tumors through the mechanism of miR-204-5p affecting tumor development (Yang et al. 2020; Cheng et al. 2021).

The same inhibitory effect on the proliferation and invasion of tumors has also been explored in renal cell carcinoma tissue samples and cell lines and cholangiocarcinoma (Lu et al. 2020; Wu et al. 2020). In addition to regulating tumor progression, it also affects the resistance of tumor cells to chemotherapy (Yao et al. 2020). MiR-204-5p inhibits the proliferation and invasion of tumor cells and increases the sensitivity of chemotherapy by downregulating RAB22A (Yin et al. 2014).

### 3.7. MiR-221-3p

MiR-221-3p is an important gene for maintaining metabolic homeostasis, and its expression changes affect liver energy metabolism. It is also an important regulator of drug resistance to various cancer treatments. MiR-221-3p is involved in energy transport and synthesis of novel fatty acids. In a study of animal hibernation, in which the body continues to function normally, the increased expression of miR-221-3p and miR-222-3p was detected by a quantitative analysis method. The regulation of lipid synthesis and metabolism by miR-221-3p may occur through affecting the role of fatty acid synthase (Nishida et al. 2021). MiR-221-3p has a certain association with metabolic diseases, and although it has an effect on adipocyte differentiation, the specific mechanism has not yet been clarified. It was found that the expression of miR-221-3p inhibited adipocyte differentiation, reduced TG storage, and also inhibited the production of new lipids. Overexpression of miR-221-3p inhibited lipid storage and adipocyte differentiation (Ahonen et al. 2021).

MiR-221-3p is an miRNA with different expression, depending upon whether it is involved in glycolysis or lipid metabolism. Especially in the absence of oxygen, miR-221-3p plays an important role in the regulation of glycolysis and lipid accumulation (Sun et al. 2020). MiR-221-3p affects cell proliferation and invasion for a variety of tumors. It was found that in liver cancer, miR-221-3p enhanced the metastasis and proliferation of liver cancer cells by targeting axis formation inhibitor 2. Cell transcription test results confirmed that the expression level of miR-221-3p was upregulated in liver cancer, affecting the growth of tumors (Dong et al. 2019). The expression of the PDZ domain containing ring finger 4 (PDZRN4) was inhibited in colon cancer, and the expression of miR-221-3p was negatively correlated with the expression of PDZRN4. The mRNA and protein of PDZRN4 were significantly reduced in the altered colon cancer tissues compared with the non-cancerous colon tissues. The increased expression of miR-221-3p significantly affected the proliferation and differentiation of colon cancer cells (Liu & Xing 2019).

The same phenomenon was also found in non-small cell lung cancer (NSCLC), in which miR-221-3p was highly expressed. Additionally, the growth and invasion of tumor cells were significantly inhibited when miR-221-3p was downregulated. Studies have shown that miR-221-3p affects the growth of NSCLC cells by targeting cell cycle regulator p27 (Yin et al. 2019). In addition to its effect on tumor growth, miR-221-3p also plays a role in the regulation of oncological drug resistance. Upregulated expression of miR-221-3p has been found in thyroid cancer, and the overexpression of miR-221-3p leads to a decrease in radiosensitivity during treatment. Therefore, the targeting of the suppressor of cytokine signaling 3 (SOCS3) gene by miR-221-3p is a potential direction that could be developed to influence the tumor sensitivity of therapeutic drugs (Ye et al. 2021). In addition to the therapeutic method, resistance to chemotherapy is also an important obstacle to cancer treatment. The downregulation of miR-221-3p reduced the sensitivity of non-small cell lung cancer to paclitaxel, and conversely, the overexpression of miR-221-3p can regulate the p53 signaling pathway and reverse the paclitaxel resistance (Ni et al. 2021). With its varied roles, miR-221-3p provides many potential targets for clinical therapy, and it also plays an important role in lipid metabolism and the regulation of tumor growth process.

Many genes involved in tumor glucolipid metabolism exhibit significant effects, and regulate tumor proliferation, invasion, metastasis, and metabolism to varying degrees (as shown in Table 1). Because each gene exists in different regulatory pathways, the extension and discovery of regulatory mechanisms and their effects are also seen as potential research directions, such as drug resistance regulation, which is considered to be an important goal of future drug resistance research.

**Table 1.** Summary of micro-RNA regulation of abnormal glucose and lipid metabolism.

Micro-RNA	Regulatory mechanism	Application development	Reference
MiR-122	MiR-122 specifically binds to APOBEC2 mRNA to inhibit expression	MiR-122 affects GLUT1 and other pathways to inhibit tumor growth	[61],[62],[63]
MiR-212-5p	MiR-212-5p inhibited lipid generation and accumulation by targeting fatty acid synthases and stearyl co-enzyme A desaturase 1	MiR-212-5p is an important inhibitor target to suppress tumor metastasis and proliferation	[66],[67],[69],[70]
MiR-27b-3p	MiR-27b-3p regulates lipid metabolism mainly by influencing the mechanism of PPAR gamma	MiR-27b-3p downregulation reverses the progression of tumors and is an important research point in the regulation of drug resistance of tumor cells	[72],[73],[75],[78],[79]
MiR-107-5b	MiR-107-5b affects the metabolic distribution of glycolipids by regulating the production of steroids	MiR-107-5b targets EGFR to inhibit tumor invasion and proliferation	[81],[83],[85]
MiR-130b	MiR-130b regulates lipid metabolism, fatty acid degradation, glucose metabolism, and other pathways	MiR-130b regulates metastasis, invasion, and proliferation of cancer cells depending on the negative correlation with HHEX expression	[87],[90],[91]
MiR-204-5p	MiR-204-5p affects lipid synthesis by affecting adipose stem cell differentiation	The negative correlation between miR-204-5p and SNHG4 can be used as a potential target for the treatment of cancer	[100],[102],[104],[105]
MiR-221-3p	MiR-221-3p acts on fatty acid synthases to regulate lipid metabolism in the body	MiR-221-3p regulates drug resistance in the body and is also involved in tumor cell proliferation	[109],[111],[112],[115]

These are just a portion of the corresponding micro-RNAs discovered at the present stage, and there is still a large number of relevant genes to be discovered. Currently, the gene regulation mechanism remains unclear, especially with single genes that may affect multiple pathways. This increases the diversity and uncertainty of confirming the regulatory role of genes. With the development of analysis and verification technology, the regulatory mechanisms of the genes related to glucose and lipid metabolism will be gradually clarified, which will also provide a theoretical basis for the design of specialized target therapy in the future, and increase the huge possibility for the treatment of cancer diseases.

## 4. Gene vectors designed to regulate glucose and lipid metabolism

With the maturity of RNA interference technology, silencing the corresponding regulatory genes of diseases by small interfering RNA (siRNA) has become a new treatment approach with great therapeutic potential, especially for diseases with many gene mutations such as tumors (Zhang & Yang 2020). However, gene drugs need to enter the mutated cells to have an effect, and it is difficult for them to reach the affected cells and be enzymatically hydrolyzed when using the normal oral or injection route. On this basis, how to deliver mRNA or siRNA into the body and to the disaffected tissues and even to the disaffected cells has become one of the obstacles in clinical treatment (Shao-Pu 2010). Viral carriers such as lentiviruses, adenoviruses, and adeno-associated viruses are traditionally used for the delivery of gene drugs (as shown in Table 2) (Safinya 2004). The virus has a broad spectrum of carrier series with a wide range of applications, and also possesses high efficiency of infection at the same time. Therefore, different virus carrier classes are suitable for the different sizes of gene fragments. However, there is a great demand for new viral vectors with high safety, strong carrying capacity, and high bioavailability (Gupta et al. 2021). To solve this problem, ordinary nanoparticles used as a drug delivery system can be wrapped with genetic drugs such as siRNA fragments. Based on the electronegativity of gene drugs, a cationic nanomedicine delivery system was constructed to adsorb gene drugs on nanoparticles through physical electrostatic action to realize packaging and transportation (Ross & Ofri 2021). Next, we summarize some vectors for the delivery of genes regulating glycolipid metabolism (as shown in Table 3), briefly elucidate their mechanisms of action, and briefly discuss their prospects for development in the future. We will also describe in detail the representative progress in the ongoing development of gene vectors for glucose and lipid metabolism, from the construction of simple gene vectors, to the construction of endogenous materials into the vector, the co-delivery of chemical drugs and gene drugs, and the exploration of the combination of gene drugs and immunotherapy.

**Table 2.** Summary of the application information of several viral vectors, such as application and safety.

Virus delivery system	Adenovirus	Lentivirus	Adeno-associated virus
Viral genome	Double-stranded DNA virus	RNA virus	Single-stranded DNA viruses
Replication	Replication-conditional	Non-replication	Replication-conditional
Virus titer	Maximum 10^12^ PFU/ml	Maximum 10^9^ TU/ml	Maximum 10^12–13^ v.g/ml
Infected cell type	Infecting dividing and non-dividing cells	Infecting dividing and non-dividing cells	Infecting cells do not divide as well
Expression degree	High expression	Moderate expression	High expression
Start time of expression	Quick (1–2 days)	Slow (2–4 days)	Slower (1–2 weeks)
Duration of expression	Viral genome is free from the host genome, and immediately expresses the exogenous genes	Viral genes are integrated into the host genome, and express foreign genes stably for a long time	Viral genomes are isolated from host genomes, and can be expressed for a long time in cells with low division
Cloning capacity	Expresses exogenous fragments less than 5 kb	Expresses exogenous fragments less than 4 kb	Expresses exogenous fragments less than 2.8 kb
Immunogenicity	High immunogenicity	Low immunogenicity	Minimal immunogenicity
Security	Causes coughing and runny nose	No pathogenicity has been found	No pathogenicity has been found

**Table 3.** Summary of current gene vectors targeting glycolipid metabolism, including the genes involved, the types of tumors used, and the effects.

Gene	Vector	Cancer	Application effect	Carrier type	Ref
MiR-122	Adeno-associated virus	Liver cancer	Significantly affect the process of tumor proliferation	Viral	(Thakral & Ghoshal 2015)
MiR-7	Lentivirus	Pancreatic caner	Impair autophagy-derived pools of glucose to suppress pancreatic cancer progression	Viral	(Gu et al. 2017)
SH-DX2	Lentivirus	Lung cancer	Suppresses Lung Cancer Cell Growth through blocking glucose uptake	Viral	(Chang et al. 2012)
TRAIL	Adenovirus	Prostate cancer	Induce cancer cell apoptosis by depleting cholesterol of lipid rafts	Viral	(Liu et al. 2015)
GRP94	Adenovirus	Colon cancer	Regulate glucose uptake to enhance cancer radiation therapy	Viral	(Liu et al. 2005)
MiR-130	Exosomes	Breast cancer	Suppress breast tumor cell invasion and migration	Non-viral	(Moradi-Chaleshtori et al. 2021)
MiR-122	LNP-DP1(cationic lipid nanoparticle)	Liver caner	Inhibit the growth rate of tumor cells by nearly 50 percent	Non-viral	(Hsu et al. 2013)
MiR-212	Chimeric peptide-condensed nanoparticle	Pancreatic ductal adenocarcinoma	Enhance the sensitivity of tumor cell to doxorubicin	Non-viral	(Chen et al. 2019)
MiR-221	polyethyleneimine-capped silver nanoclusters (PEI-AgNCs)	Liver cancer	Regulate lipid metabolism to increase the sensitivity of drugs and exhibit the effect of bacterial inhibition	Non-viral	(Du et al. 2017)
siGRP78	DOTAP (1,2-dioleoyloxy-3-trimethylammoniumpropane) liposomes	Breast cancer	Increase the sensitivity of tumor cell to chemotherapy	Non-viral	(Samson et al. 2019)

Salt inducible kinase 1 (SIK1) plays an important role in the regulation of glucose and lipid metabolism, especially in the process of liver metabolism. SIK1 expression also shows an important effect on metabolic diseases (Hartono & Lee 2018). In diabetes studies, under the condition of high glucose, SIK1 expression will be downregulated, which will further affect the metabolic process of liver gluconeogenesis. Under the abnormal state of this process, insulin resistance will be produced, which will affect the treatment of diabetes (Wang et al. 2020). Based on the above mechanism, adenovirus transduction constructed by Song et al. induced high expression of SIK1. The upregulation of SIK1 expression affects glucose metabolism, the expression of lipid genes, the overall metabolism of the body, and changes in manifestations, in some cases (Song et al. 2019).

The recombinant adenovirus was constructed by cloning the 2,337 base-pair PCR product into linearized adenovirus plasmid GV314 using T4 DNA ligase and transfecting into competent *Escherichia coli* cells. Positive clones were screened by ampicillin resistance and then underwent ABI 3730 sequencing analysis. SIK1-overexpressing adenovirus (Ad-SIK1) was packaged in human embryonic kidney 293 T cells and purified with the Adenovirus X purification kit. The virus titer was determined by an end-point dilution method (Zhou et al. 2019). The results showed that SIK1 plays an important role in the regulation of liver glucose and lipid metabolism, and it inhibits liver gluconeogenesis and lipogenesis. SIK1 also plays a role in the regulation of metabolic diseases and is found in tumor diseases. In cervical squamous cell carcinoma cells, SIK1 inhibits the invasion and metastasis of cancer cells (Peng et al. 2020). In colorectal cancer, the upregulated targeting of SIK1 by miR-17 has been found to promote the process of colorectal cancer, and thus, this mechanism has also become a potential therapeutic target (Huang et al. 2019).

**Figure 3.**
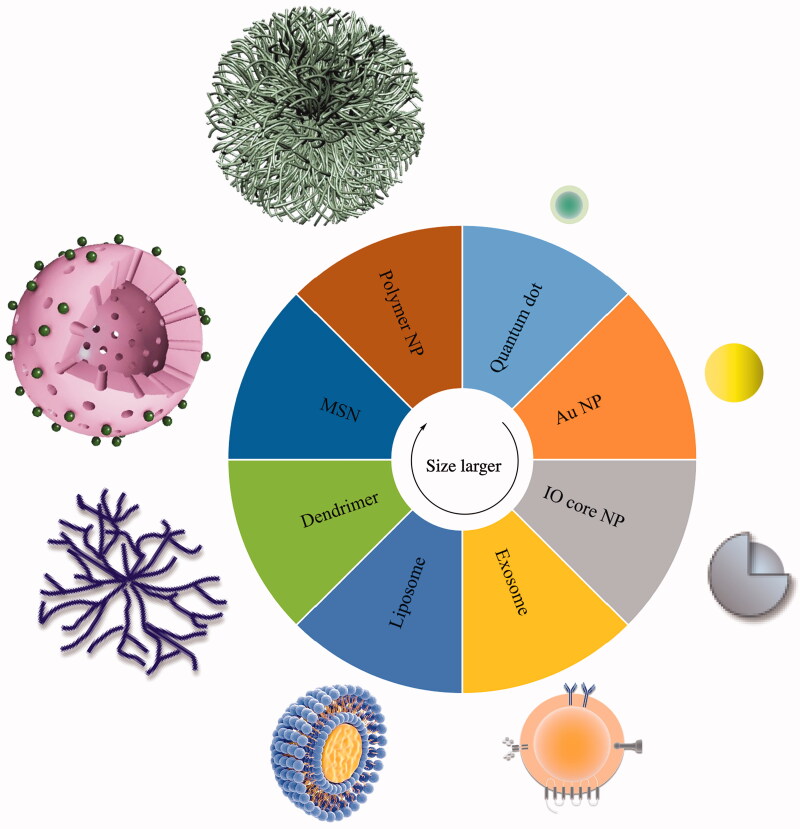
Summary of gene vectors constructed from various materials.

In addition to traditional viral carriers, there are other types of non-viral carriers that are suitable. Exosomes, metal nanoparticles, inorganic materials, bioorganic materials, and polymers have been gradually used for the construction of nano-gene carriers (as shown in Figure 3) (Boca et al. 2020; Yan et al. 2020). Next, we will introduce cases where several materials have been applied.

**Figure 4.**
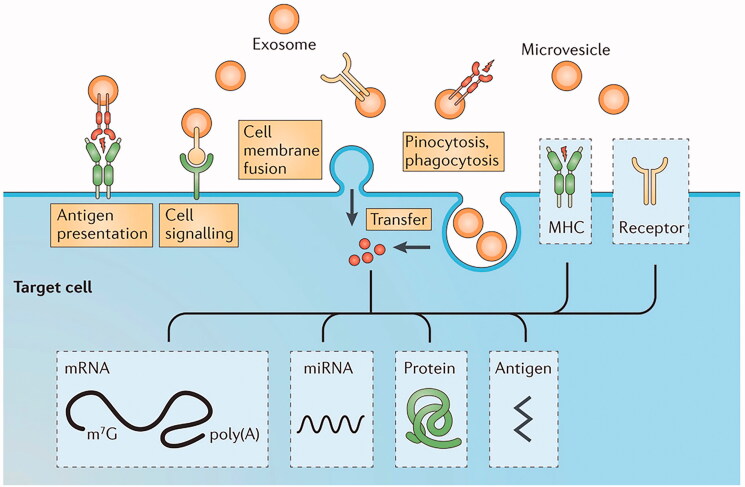
Schematic diagram of exosomes entering targeted cells. Exosomes can enter cells through a variety of signaling pathways, and their contents include mRNA, proteins, and antigens. This figure has been adapted/reproduced from ref 135 with permission from John Wiley and Sons.

Exosomes are small membranous vesicles containing complex RNA and proteins with a diameter range of 40–100 nm and can be secreted by a variety of cells. Exosomes were considered as a vehicle for transporting metabolic waste when they were initially discovered. Exosomes have gradually been found to participate in many physiological processes, and they are considered to have great potential research value because they are able to deliver RNA and protein substances (Ge et al. 2017; Hashemian et al. 2020). Exosomes are rich in cholesterol and lecithin, which have satisfactory biological activity. Additionally, exosomes can capture the mRNA secreted by human cells in vitro and translate it into protein, indicating that the mRNA transferred by the exosomes has biological activity and can regulate the mRNA level of target cells (as shown in Figure 4) (Yang et al. 2019). Thus, there is increased research on the use of exosomes as gene carriers and for the delivery of gene drugs (Vojtech et al. 2014; Silva and Melo 2015).

Exosomes are an endogenous substance in the body, and therefore, the acquisition of exosomes is often accomplished by the separation of macrophages, rather than physical or chemical methods (Familtseva et al. 2019; Pegtel & Gould 2019). Tian et al. established a high-glucose mouse model to observe the performance of mice, and added high-glucose medium-induced macrophages to RAW264.7 macrophages in culture. After the culture, the exosomes were obtained by centrifugation (Lasser et al. 2012). The expression of miR-210 in exosomes was detected by RT-PCR and western blot analysis, and the reduction in glucose uptake was also experimentally verified (Tian et al. 2020). The total amount of exosomes in tumor tissues was significantly higher than that in normal tissues.

Studies have found that exosomes play a certain regulatory role in the process of tumor growth, such as affecting the proliferation and metastasis of tumor cells (Li et al. 2019; Zhang & Yu 2019). Pan et al. constructed exosomes of miR-130b and miR-130b-mv to inhibit PPAR-gamma through translation in an obese mouse model induced by a high-fat diet, and it was observed that epididymal fat deposition was reduced, and glucose tolerance was partially restored (Pan et al. 2015). Another experiment in breast cancer (4T1 cells) showed that miR-130 loaded by exosomes significantly inhibited the invasion and metastasis of tumor cells (Moradi-Chaleshtori et al. 2021). Based on the abnormal manifestations of exosomes in the body of tumor patients, exosomes or exosomal microRNAs can be used as an important detection standard, providing the possibility of early tumor detection (Nedaeinia et al. 2017; Chen et al. 2019).

As an endogenous transport carrier, exosomes exhibit satisfactory biocompatibility, which can reduce the immune response and increase the circulation time of drugs in the body. An efficient nano-drug delivery system can be created with exosomes that are loaded with therapeutic gene or protein drugs that target cells (Kalluri & LeBleu 2020). However, there is greater difficulty in the process of exosome extraction, and it still faces some technical challenges. Low doses of exosomes do not appear to induce a strong immune response, but the effect of immune rejection needs to be confirmed in further experiments (Batrakova & Kim 2015). More importantly, as an exosome outside a secretion, it is a complex and lengthy process to induce macrophages to regulate changes in their expression. Much time and energy must be consumed for large-scale preparations because of the difficulty of the process (Luan et al. 2017).

**Figure 5.**
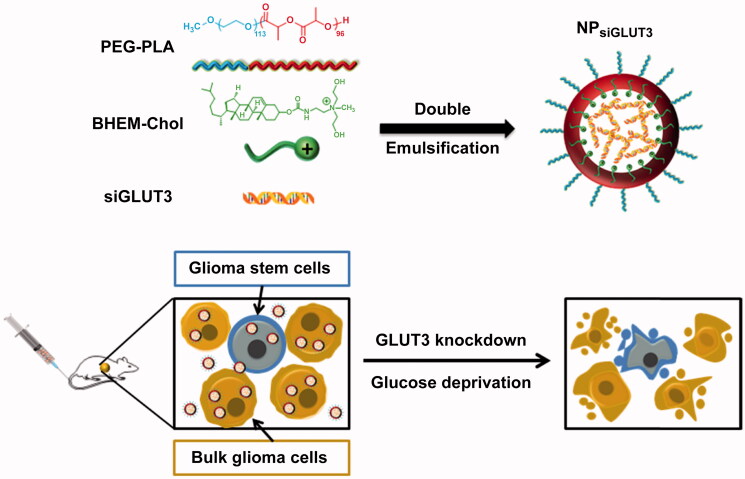
Schematic diagram of NP_siGLUT3_ synthesis and its mechanism of action in the body. This figure has been adapted/reproduced from ref 152 with permission from ELSEVIER, Copyright 2015.

With the development of polymer materials, there has been considerable pharmaceutical research on nano-drug delivery systems for gene drugs, especially by using the electrostatic adsorption of cationic materials and genes to carry gene drugs into the body. Polymeric vectors are safer alternatives for gene delivery because of their advantages as compared to viral vectors (Liu et al. 2021; Yan et al. 2021). Xu *et al*. used polymer materials such as polyethylene glycol, polylactic acid, and the cationic lipid BHEM-Chol to form a diblock copolymer. This material was able to emulsify and encapsulate gene drugs in siRNA aqueous solution to form homogeneous nanoparticle gene carriers that were called NP_siGLUT3_ (as shown in Figure 5) (Xu et al. 2015). Given every other day for a sustained period of time, the tumor volume of the glioma (U87MG cell) mouse model was effectively controlled compared to the blank group. There was a significant inhibitory effect on metabolism in the experimental group injected with NP_siGULT3_.

The nanocarriers constructed from polymer materials possessed satisfactory stability, and the presence of polyethylene glycol (PEG) prolonged the retention time *in vivo*. siRNA exhibited satisfactory gene-loading capacity through electrostatic adsorption with cationic lipids. In addition to the inhibition of metabolism, there were also certain inhibitory effects on the proliferation and differentiation of tumor cells (Yang et al. 2011; Li et al. 2014).

**Figure 6.**
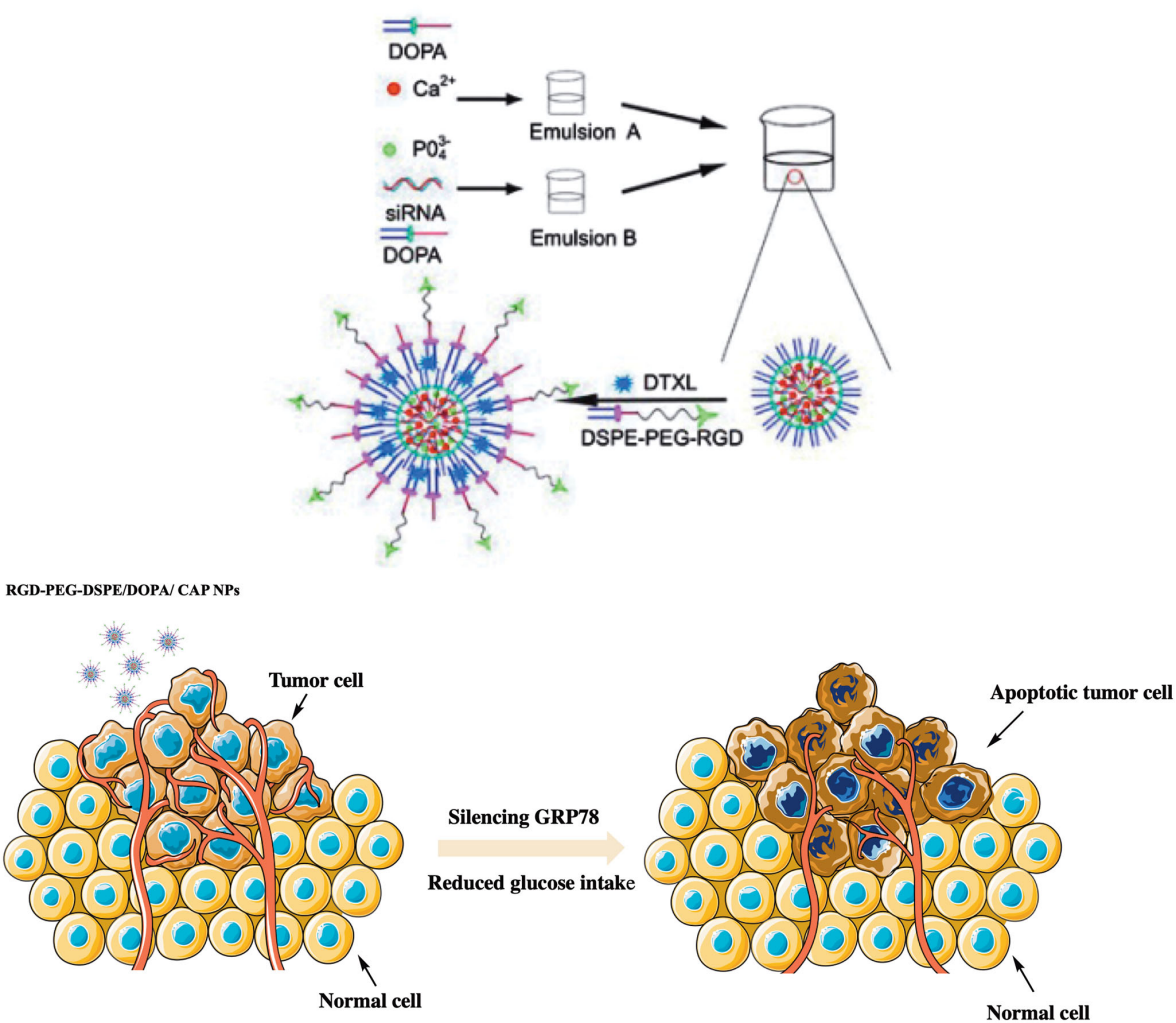
Schematic diagram of the construction of RGD-PEG-DSPE/DOPA/CAP nanoparticles, and mechanism of action of the RGD-PEG-DSPE/DOPA/CAP nanoparticle tumor treatment. This figure has been adapted/reproduced from ref 155 with permission from Dove Medical Press, Copyright 2019.

RGD is a neovascularization-targeting peptide that can provide a satisfactory targeting effect; PEG provides good biocompatibility and prolongs the action time *in vivo*; and distearoyl phosphoethanolamine (DSPE) provides good lipid solubility, which facilitates the crossing of biological barriers. Therefore, there has been considerable research on the novel diblock copolymer RGD-PEG-DSPE. The one constructed by Zhang et al. contained a core of calcium phosphate (CaP), and it was highly efficient at loading siRNA, while its dioleoyl phosphatidic acid (DOPA) and RGD-PEG-DSPE components are excellent for loading chemical drugs (Zhang et al. 2019) (as shown in Figure 6). The co-loading of gene drugs and docetaxel produced a satisfactory synergistic effect on PC (prostate cancer)-3 cell line. The experimental group co-loaded with DTXL and siRNA significantly improved the sensitivity of chemical drugs, and tumor cells also showed many positive changes in proliferation and apoptosis. Chemical drug and gene drugs total load, which reduce the drug dosing frequency. After gene regulation, chemical drugs can play a role in reversing tumor cells for the sensitivity of drug, even compared to before enhancement effect, changed the significant problem of chemical drug resistance.

GRP87 is a glucose-regulated protein that plays a key role in tumor cell survival, tumor progression, metastasis, and resistance to therapy. The upregulation of GRP87 expression is beneficial for the continuous adaptation of the endoplasmic reticulum (ER), which can improve glucose metabolism (Ni et al. 2011; Lu et al. 2020). The expression of GRP87 was silenced by siRNA technology, and the glucose metabolism of the body was decreased. The particles also effectively reversed multidrug resistance and inhibited tumor proliferation and metastasis (Gifford & Hill 2018). Of course, the inorganic material added will adsorb siRNA and produce drug-loading effects. Compared with the polymer, its toxicity is greatly reduced, and the biological compatibility is increased.

Mesoporous silica nanoparticles are also commonly used as drug delivery carriers. Due to their satisfactory drug delivery efficiency and release ability, they have been used with a variety of drug preparations. Shi *et al*. constructed a gene-loaded nanocarrier with controlled release. After the mesoporous silica was loaded with chemical drugs, MDR(multi-drug resistance)-1 siRNA was adsorbed through the physical adsorption capacity of the material. As a well-known drug resistant protein, MDR-1 also plays an essential role in the regulation of lipid formation pathways(Yun et al. 2013). Then, hyaluronic acid was coated on the outermost layer, and this enabled the siRNA to avoid decomposition and deactivation by the corresponding enzymes for increased efficacy (Shi et al. 2019).

**Figure 7.**
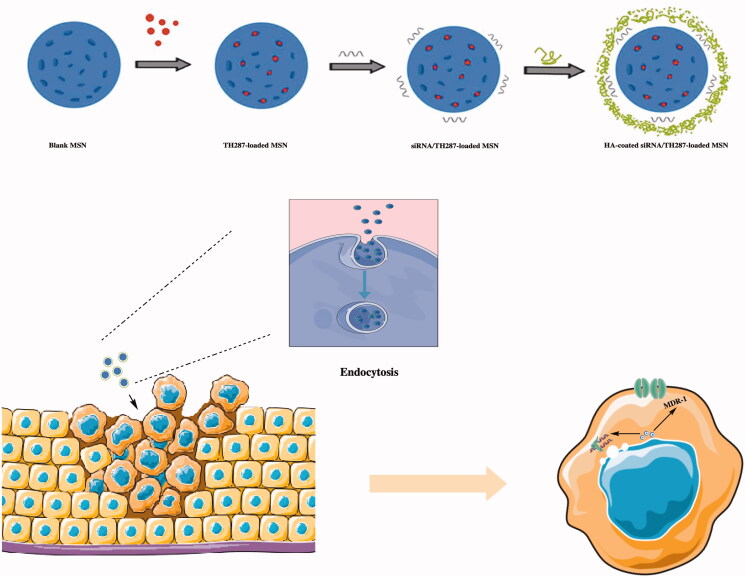
Preparation of HA-coated MDR1 siRNA/TH287-loaded MSN, and the mode of action for MDR1 siRNA/TH287-loaded MSN.

When nanoparticles enter into tumor cells by endocytosis, TH287(MTH1 inhibitor) can damage DNA, which decreases tumor cell proliferation. Subsequently, there is reduced MDR-1 small interference RNA function, which greatly improves the therapeutic effect. According to this model, this will regulate glucolipid metabolism-related genes via the gene drugs and/or chemicals that were carried. The delivery system of HA-siTMSN make great progress on treating oral squamous cell carcinoma (OSCC), the HA-siTMSN drug delivery system showed the strongest tumor inhibition in a tumor model constructed from CAL27 cells in mice. To avoid the low efficacy of a single pathway, a mesoporous carrier for inorganic materials is an optimal choice because its application cost is low, and it also exhibits satisfactory biological safety and high plasticity that enables the nature of the carrier to be changed with only small amounts of modification. Because of all these advantages, it is a drug carrier with great potential (as shown in Figure 7).

**Figure 8.**
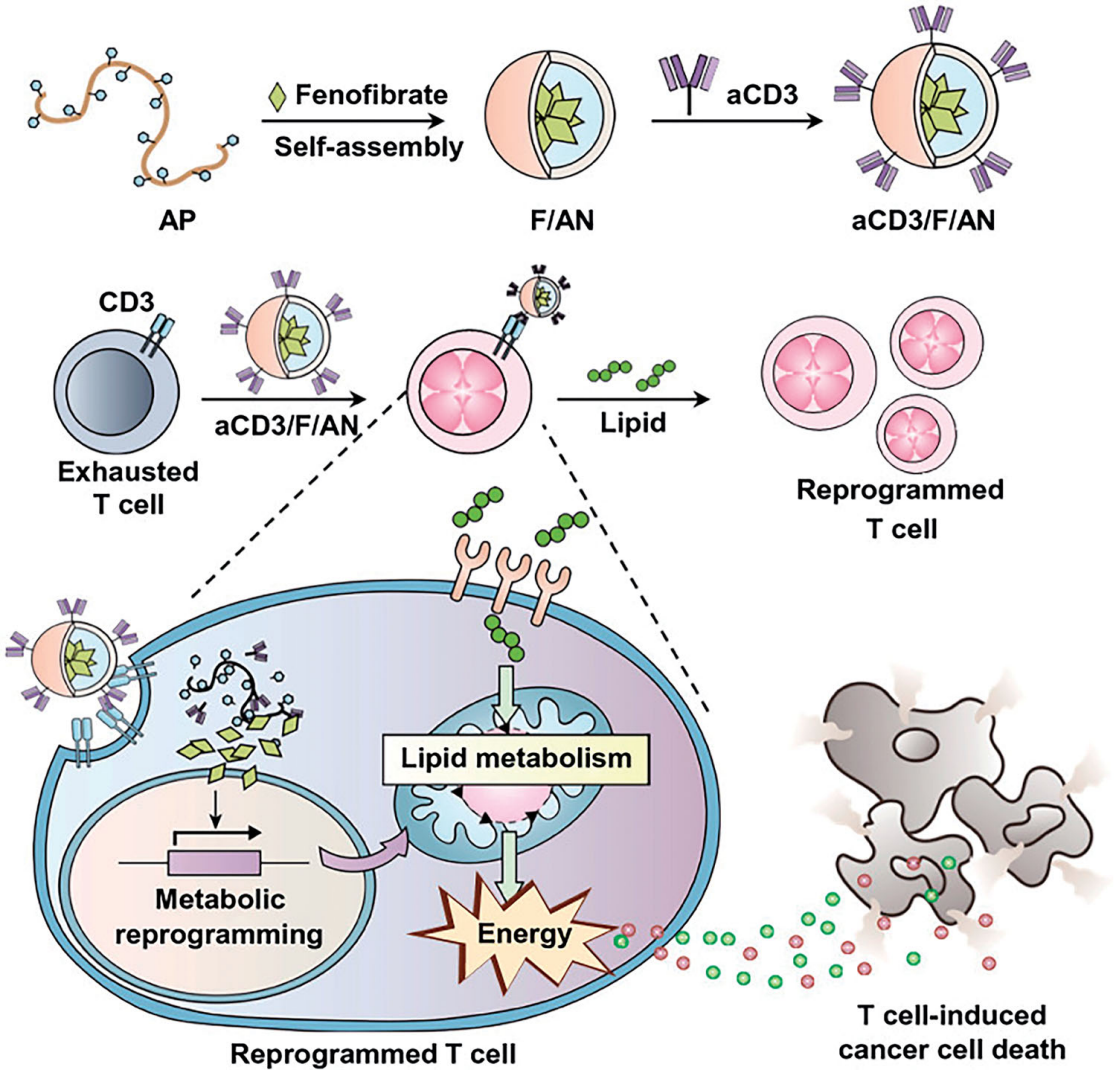
Schematic diagram of the preparation process of aCD3/F/AN and its mechanism of action in vivo. This figure has been adapted/reproduced from ref 161 with permission from Springer, Copyright 2021.

With the continuous exploration of tumor treatment, immunotherapy has gradually become the focus of research. To improve the therapeutic efficiency of a single drug delivery system, the delivery system and immunotherapy have been combined (Yan et al. 2021). In addition to the common carriers for drug delivery, a nanocarrier with the ability to activate and regulate T cells can be constructed through an antibody-modified nanosystem to kill tumor cells. It subverts the traditional treatment method that uses nanometer drug delivery systems. Kim et al. used amphiphilic polyglutamic acid to encapsulate fenofibrates into micelles by self-assembly (as shown in Figure 8). The anti-CD3E F(Ab')2 fragment was attached to the surface of the micelles, and finally, micelles with anti-CD3-modified drugs were formed. When injected into the body, these micelles can bind to T cells, enter T cells, alter the regulation of metabolism, and activate and promote the synthesis of fatty acids in the body. The synthetic fatty acids provide energy for the proliferation of T cells in the absence of glucose in the tumor, and then, T cells induce the apoptosis of cancer cells to achieve the effect of cancer treatment (Kim et al. 2021). The construction of the delivery system provides a good perspective for the combination of drugs targeting glycolipid metabolism genes. This also provides theoretical support for the construction of vector for co-loading gene drugs targeting glycolipid metabolism and induced immunotherapy in the future.

The micelle carrier prepared by polymer materials, combined with immunotherapy, resulted in modified antibodies on the surface of the carrier to achieve a targeting effect. Then, the carriers were able to successfully enter into T cells, regulate the lipid metabolism process, and guide autoimmune T cells to induce cell apoptosis. The drug delivery system reduced the side effects of drugs, and maximized the immune function of the body through internal regulation.

## 5. Discussion and prospects

The rapid development of gene technology provides a powerful theoretical and technical basis for gene therapy, which requires the construction of efficient gene carriers, and provides the appropriate genetic drugs and a large amount of genetic data. However, exploring the mechanisms of gene regulation is just the tip of the iceberg in the quest to affect the regulation of the entire body, especially when taking into account the complexity of genes, which causes more obstacles and challenges for gene therapy. Of course, with the continuous innovation of genetic engineering technology, the speed of gene screening steadily increases and becomes timelier, providing more optimal gene selections for the preparation of suitable gene drugs.

Based on the abnormal metabolic environment of a tumor, by regulating metabolic genes, abnormal glucose and lipid metabolism in tumors can be affected, the development process of tumors can also be affected, and drug resistance in tumor therapy can then be reversed. The synergistic therapeutic effect generated by co-loading with chemotherapy drugs plays a significant role in clinical treatment.

Furthermore, in the preparation of gene vectors, a variety of vector forms have been discussed. The biological carrier possesses satisfactory biocompatibility, but there are high requirements for its extraction technology, the process is more complicated, and the low yield is also a problem worthy of discussion. The physical drug carrier, through its unique physical properties, can efficiently release a drug, and a highly efficient drug delivery system is created; if biological barriers exist, it will be difficult to efficaciously treat the internal tumor.

In addition, polymer materials are being rapidly developed and more widely used in biomedical applications, and the appropriate polymer materials can provide cationic properties and generate electrostatic adsorption on RNA to achieve the purpose of delivering gene drugs. Polymer materials have high plasticity and can be used to synthesize many functional groups through chemical reactions, which will result in increased targeting and prolonged circulation in the body. The drug delivery system will exhibit some characteristics of environmental response due to the presence of some chemical bonds.

With the interaction and integration of various disciplines, the application of drug delivery systems in the field of gene delivery is becoming increasingly thorough, which has broad clinical application prospects and great potential for the diagnosis and treatment of diseases. However, there remains much work that is necessary to prepare novel gene vectors and to continue to find and create additional nanotools for gene therapy.
